# Newborn Care: What We Can Learn from the Kangaroo Mother

**DOI:** 10.3389/fpubh.2014.00096

**Published:** 2014-07-24

**Authors:** Donald E. Greydanus, Joav Merrick

**Affiliations:** ^1^Department of Pediatric and Adolescent Medicine, Western Michigan University Homer Stryker MD School of Medicine, Kalamazoo, MI, USA; ^2^National Institute of Child Health and Human Development, Jerusalem, Israel; ^3^Health Services, Division for Intellectual and Developmental Disabilities, Ministry of Social Affairs and Social Services, Jerusalem, Israel; ^4^Division of Pediatrics, Hadassah Hebrew University Medical Center, Jerusalem, Israel; ^5^Kentucky Children’s Hospital, University of Kentucky College of Medicine, Lexington, KY, USA; ^6^Center for Healthy Development, School of Public Health, Georgia State University, Atlanta, GA, USA

**Keywords:** newborn, neonatology, pediatrics, human development, kangaroo method, kangaroo mother care, skin-to-skin care

## Introduction

The word “kangaroo” comes from the Guugu Yimithirr word “gangurru,” which refers to gray kangaroos [*Macropus fuliginosus* (Western); *Macropus giganteus* (Eastern) (1)]. The name was first written as “Kanguru” on July 12, 1770 by Sir Joseph Banks (1743–1820), who was with the famous Captain James Cook (1728–1779) at the modern Cooktown in Australia, while they were repairing their ship – the HMS Endeavour damaged on her historic voyage to the Great Barrier Reef (2). The language of the indigenous people of Cooktown is Guugu Yimithirr.

The brobdingnagian joey (infant kangaroo) crawls over the mother kangaroo’s fur in approximately 3 min to arrive at the mother’s pouch, which contains four teats to feed the baby; milk of different chemical compositions are provided to the esurient joey by the different quad-teats (3, 4). The mother kangaroo seems to have little interest in her newborn at first, but the joey stays in the pouch feeding and growing – eventually leading to a powerful, henotic bond between mother and baby. The young kangaroo joey first absquatulates the pouch for a few minutes at 198 days of life and then abscises at an average of 235 days at an average weight of 4–5 kg. Though outside the mother’s pouch, the baby (infant) will suckle for another 4 months until a year of age. This joey has a close vinculum with its mother for sometime thereafter due to the healthy touching that the pouch-suckling milieu created (3, 4). Koala (*Phascolarctos cinereus*) mothers have developed the same feeding technology over the millions of years of their evolution leading to a successful bond between this mansuetudine mother and her bonhomie baby who like all marsupial newborns is called a joey (5, 6).

Though the kangaroo mother figured out the best way to feed and bond with her joey millions of years ago, it took *Homo sapiens* much longer to learn about caring for its newborn and to be taught this “kangaroo” care (7). Modern neonatology has developed over the past few centuries and has acquired amazing technology in the twenty-first century (7). However, it was not until 1978 that Dr. Edgar Rey Sanabria, professor of neonatology, established “kangaroo care” for premature and low-weight newborns in Bogotá, Colombia; his aperçu on premature infants was in response to overcrowding and lack of resources in his hospital (8, 9). Professor Sanabria received the World Health Organization (WHO: Geneva, Switzerland) Sasakawa Health Prize in 1991 for his idea and work suggesting that physical closeness between the premature newborn and the mother in a skin-to-skin contact might make up for lack of modern newborn services and resources such as incubators. Indeed, if it worked for marsupials and other animals for millions of years, how about the human newborn?

## Skin-to-Skin Contact

Three main elements of kangaroo care (koala stereotropism) have emerged: first, continuous skin-to-skin contact is thermal care (thigmatropism) to maintain the premature baby’s core temperature; second, there is encouragement of exclusive breastfeeding with all the known ferocious benefits of lactation. Finally, there is stereotropism-induced colonization of the preemie with the mother’s commensal microorganisms to protect the immunologically immature baby from nosocomial infection (10).

This method of newborn care was promoted by the World Health Organization that published a guide for its use in 2003 (11). Its actual acceptance over the years by various professional groups of dubiety consider it to be an adiaphorous method as some studies may fail to find reduction in mortality or other benefits already known for millions of years by marsupials (12). Other studies, however, have found beneficial effects from this method and it has been used by various newborn units and newborn intensive care units around the world (10). An article by Suzanne Rutgers Greydanus and Sheryl Meyers (13) provides more details on this method of newborn care adopted from the famous Australian marsupial named in 1770 from the Guugu Yimithirr language native to Cooktown, QLD, Australia.

As researchers study the exact benefits of kangaroo mother care (KMC), one issue behavioral scientists point out is the importance of touch for normal health in both humans and non-human animals. Human skin has evolved over millions of years of life’s development from a simple envelope to cover the ancient microorganisms to the complex covering of humans we call skin (14). The skin is a barrier needed for human existence and consists of the epidermis [stratum corneum, stratum lucidum (palms, soles), stratum granulosum, stratum spinosum, stratum basale/germinativum), and the dermis (stratum papillare and stratum reticulare] (15). The impenetrable skin and the complex central nervous system have evolved in a concinnous manner over millions of year together and also develop *in utero* for each human from the beginning of the human’s embryological development (14).

This delicate démarche or dance between the largest organ of the body (the sanguineous skin) and the intricate central nervous system (including the sympathetic and parasympathetic nervous systems) has profoundly felicitous effects on the medical and psychological health of the human being from birth to death. Research has shown that touch is an important aspect of allowing as well as promoting normal growth and development in both animals [i.e., roundworm larvae (*Caenorhabditis elegans*), rat pups (*Rattus norvegicus*)] and human infants (*H. sapiens* infants) (16). Thus, one can *a priori* speculate that the kangaroo skin-to-skin method will provide important touching sensation between mother and baby that is critical for vital cellular and molecular mechanisms leading to improved medical and psychological health in the vulnerable newborn (14, 16–19). Such touching is also beneficial for the mother to relieve her anxiety, act as a nepenthe, and increase bonding with her beloved yet precarious, premature, abecedarian, and premature catechumen (20).

## Conclusion

Sensory stimulation is important for normal development in lumbricine-, murine-, macropine-(macropodine-) animals as well as humans. As the human being develops throughout childhood, adolescence, and adulthood – touch (i.e., massage, hebetic-initiated erotic touching, and others) remains essential to normal human health. The kangaroo and koala learned this millions of years ago, but humans in the twenty-first century have finally caught on (there is nothing new under the sun as written in Kohelet/Ecclesiastes)! Life is indeed fugacious yet intimate touch is part of the foudroyant qualities of life. The A Fortiori lesson from the ancient Australian marsupials and the life they evolved from on planet earth is to maximize the amaranthine powers of touch from the first embers of life to the end until the centrifugal universe apocalyptically expands into its final eonian, Cadmean desinence (21).

A kiss may just be a kiss, a sigh may be just a sigh, but a touch can change your life (or at least your nervous system!) (16)

## Conflict of Interest Statement

The authors declare that the research was conducted in the absence of any commercial or financial relationships that could be construed as a potential conflict of interest.
